# Determination of Beta-Defensin Genomic Copy Number in Different Populations: A Comparison of Three Methods

**DOI:** 10.1371/journal.pone.0016768

**Published:** 2011-02-22

**Authors:** Peder Fode, Cathrine Jespersgaard, Robert J. Hardwick, Helen Bogle, Michael Theisen, Daniel Dodoo, Martin Lenicek, Libor Vitek, Ana Vieira, Joao Freitas, Paal Skytt Andersen, Edward J. Hollox

**Affiliations:** 1 Department for Microbiological Surveillance and Research, Statens Serum Institut, Copenhagen, Denmark; 2 Department of Clinical Biochemistry and Immunology, Statens Serum Institut, Copenhagen, Denmark; 3 Department of Genetics, University of Leicester, Leicester, United Kingdom; 4 Noguchi Memorial Institute for Medical Research, University of Ghana, Legon, Ghana; 5 Department of Clinical Biochemistry and Laboratory Diagnostics, 1st Faculty of Medicine, Charles University in Prague, Prague, Czech Republic; 6 4th Department of Internal Medicine, 1st Faculty of Medicine, Charles University in Prague, Prague, Czech Republic; 7 Department of Gastroenterology, Hospital Garcia de Orta, Almada, Portugal; Oregon State University, United States of America

## Abstract

**Background:**

There have been conflicting reports in the literature on association of gene copy number with disease, including *CCL3L1* and HIV susceptibility, and β-defensins and Crohn's disease. Quantification of precise gene copy numbers is important in order to define any association of gene copy number with disease. At present, real-time quantitative PCR (QPCR) is the most commonly used method to determine gene copy number, however the Paralogue Ratio Test (PRT) is being used in more and more laboratories.

**Findings:**

In this study we compare a Pyrosequencing-based Paralogue Ratio Test (PPRT) for determining beta-defensin gene copy number with two currently used methods for gene copy number determination, QPCR and triplex PRT by typing five different cohorts (UK, Danish, Portuguese, Ghanaian and Czech) of DNA from a total of 576 healthy individuals. We found a systematic measurement bias between DNA cohorts revealed by QPCR, but not by the PRT-based methods. Using PRT, copy number ranged from 2 to 9 copies, with a modal copy number of 4 in all populations.

**Conclusions:**

QPCR is very sensitive to quality of the template DNA, generating systematic biases that could produce false-positive or negative disease associations. Both triplex PRT and PPRT do not show this systematic bias, and type copy number within the correct range, although triplex PRT appears to be a more precise and accurate method to type beta-defensin copy number.

## Introduction

Characterization of genetic variants is fundamental in understanding human heterogeneity and susceptibility to disease. Regions where humans differ in diploid DNA dosage are known as copy number variations (CNV) and are an important component of genetic variation. CNVs are believed to encompass more nucleotide content than single nucleotide polymorphisms (SNPs) [1] and between 12% and 18% [2], [3] of the euchromatic human genome is suggested to be copy number variable [4]. The use of array-comparative genomic hybridisation (array-CGH) and next-generation sequencing techniques will probably reveal an even greater proportion of structural variation among individuals and populations [5], [6]. CNVs can alone or in combination with SNPs correlate with certain diseases, or are associated with increased susceptibility to diseases [7]–[9] including psoriasis [10]–[12], autism [13], cancer [14], schizophrenia [15]–[17], systemic lupus erythematosus [18]–[20], Alzheimer's disease [21], Charcot-Marie-Tooth disease [22], [23], Parkinson's disease [24] and autoimmunity [25].

One region showing extensive CNV is found in the 8p23.1 chromosome [26]–[28]. The region contains a cluster of defensin genes including the *DEFB4* and the *DEFB103* which encode human β-defensins 2 and 3 (hBD2, hBD3), respectively. Defensin genes encode small cationic peptides that have antimicrobial activity and have multi-functional activity: they function as chemo-attractants for T-lymphocytes, monocytes and dendritic cells [29]–[32]. β-defensins induce the production of diverse chemokines and cytokines such as MCP-1, macrophage inflammatory protein 3-α (MIP-3), RANTES, IL-6, IL-10, interferon-inducible protein 10, TNF-α and IL-1β, mainly in keratinocytes [33], [34]. hBD3 mediates monocyte/macrophage migration [35], can signal through melanocortin receptor 1 [36], and may function as an anti-inflammatory molecule [37].

The beta-defensin cluster varies in copy number between 2 and 12 copies per diploid genome with most people having 2–7 copies [28], [38]–[41]. Because, to date, all evidence suggests this defensin cluster varies in copy number *en bloc*, an assay for any point within the defensin cluster can be assumed to measure copy number across the whole region [42]. Though attention has moved from CNV discovery in small cohorts to CNV typing in larger cohorts, it is still a major challenge to determine exact copy numbers. Although several studies have been performed to characterize CNVs, comparing results from these studies has been hindered by small sample sizes and different study designs and analytical methods resulting in conflicting results [43], [44] illustrating the need for an accurate method and controls for future work. In particular, the use of real-time QPCR methods, which appear attractive due to their universal applicability, high throughput and relative simplicity, has come under some scrutiny [45], with the suggestion that batch variation can generate false positive associations of copy number and disease [46], [47]. Since CNVs may play a role in susceptibility to certain diseases, due to variation in gene expression [48], [49], it is of great importance to find approaches to determine exact copy numbers.

PRT addresses one of the major drawbacks of QPCR, namely the problem of bias due to different amplification kinetics of test and reference amplicons [50]. By careful design of primers to a repeat region within the CNV, it is possible to amplify two almost identical regions using just one set of primer pair, the test amplicon within the CNV, and the reference amplicon outside the CNV, ideally on another chromosome. A small difference in length between the amplified products distinguishes test amplicon from reference amplicon. Using PCR and subsequent fragment analysis by capillary electrophoresis it is possible to determine the copy number. Here, we evaluate a simple, cost-effective, high-throughput adaptation of the PRT method to determine gene copy number using the pyrosequencing technique to quantify sequence differences between the test and reference PRT amplicons [42], [51], [52], and compare this pyrosequencing PRT (PPRT) with the published triplex PRT [53] method and real-time QPCR. We determined the gene copy numbers for 576 healthy individuals from five different populations using these three methods, compared the copy number distributions generated by the different methods, and compared the copy number distributions across the different populations.

## Materials and Methods

### Study population

Unrelated DNA samples from normal healthy individuals from five different demographic population groups, Denmark (n = 174), Czech Republic (n = 21), Ghana (n = 100), Portugal (n = 91), and the United Kingdom (the European Collection of Cell Cultures ECACC (Cat. No.: HRC-1 and HRC-2); (n = 190) were used in the study.

### Ethics statement

All samples were gathered with full ethical consent and appropriate documentation as stipulated by the ethical consent, which normally involved written informed consent. For Portuguese samples, the local ethics committee of Hospital Garcia de Orta, Almada, Portugal, gave its approval for a genetic study using blood samples from local patients and normal volunteers. For Czech samples, the study has been approved by the Ethical commitee of the General Faculty Hospital in Prague. For Ghanaian samples, approval of the Institutional Review Board of Noguchi Memorial Institute for Medical Research, University of Ghana was given. For Danish samples, they are a standard set of anonymised DNA samples provided for standardisation purposes and quality assurance, and were not required under Danish law to pass the Danish Ethics Approval system. Other samples were from immortalised lymphoblastoid cell lines, which had been derived from B-cell lymphocytes with full ethical consent by the suppliers (ECACC and Coriell Cell Repositories).

### DNA extraction

Genomic DNA was isolated from samples of venous blood, anti-coagulated with EDTA and purified using the QIAamp DNA Blood Mini Kit (Qiagen, Hilden, Germany) according to the manufacturer's protocol and eluted in water. The UK samples were purified using an in-house magnetic bead affinity method and was obtained from ECACC (Porton Down, Salisbury, UK). Czech DNA samples were extracted using a routine “salting out” and ethanol precipitation procedure.

### Quantitative real-time PCR for copy number determination

A duplex TaqMan real-time quantitative PCR-based assay was developed for the detection of beta-defensin genomic copy number using RNaseP as reference gene. Primers and probes for amplification of a region near the *DEFB103* gene were designed using the Primer Express software, version 2.0 (Applied Biosystems, Foster City, CA). The sequences of the primers and probes were as follows: *DEFB103* forward primer (5′ CAT AGG GAG CTC TGC CTT ACC A 3′); *DEFB103* reverse primer (5′TGC AGA ACA CAC CCA CTC ACT C 3′) and *DEFB103* probe (5′ FAM - TGG GTT CCT AAT TAA C – MGB 3′). The sequence for RNaseP is not known since it is a commercial available kit (VIC labelled, Cat. no. 4316844, Applied Biosystems). The amplification efficiencies of the target genes and the reference genes were tested to be approximately equal not varying more than 5% from each other. Optimizing runs were performed to define limiting primer concentrations for the duplex assay. The PCR reactions (20 µl) were carried out in triplicate with 10–75 ng of template DNA, 1 x Brilliant II QPCR master mix (Stratagene, La Jolla, CA), 400 nM of *DEFB103* probe, 600 nM *DEFB103* primers and 1 x RNaseP primer mix. Each plate included triplicate wells of “no template control” and 4 control samples. QPCR was performed using a Stratagene MX3000P machine (Stratagene) using the following conditions 95°C for 10 min followed by 40 cycles of 95°C for 15 sec, and 60°C for 1 min. In all runs samples from Coriell Institute for Medical Research (Camden, NJ) with known copy number were included[50]: 3 copies per genome (Coriell cat no.: NA10861), 4 copies per genome (Coriell cat no.: NA07048), 5 copies per genome (Coriell cat no.: NA10846), and 7 copies per genome (Coriell cat no.: NA10847). These were used to generate a correction curve by linear regression, and corrected copy number estimates calculated for each sample calculated using this run-specific regression equation.

### Paralogue Ratio Test for copy number determination

PRT was performed as described previously [53]. Briefly, the assay used comprises two PRT assays and a multiallelic ratio test [52] to gain three independent measurements of beta-defensin repeat copy number. This test, performed in duplicate, produces six estimates of beta-defensin copy number in a single fluorescent capillary run, and each test is independently normalised against six samples of known copy number to control for variation between experimental runs. The six values are combined using a maximum-likelihood method [53], [54] to give the best estimate of the integer copy number for each sample, together with an associated significance value reflecting the confidence we have in that typed copy number compared to all other copy numbers between 1 and 10. For analyses involving raw non-integer copy number estimates, we calculated means of the six copy number estimates, each estimate weighted according to the inherent variability of each individual assay.

### Pyrosequencing-based Paralogue Ratio Test for Copy number determination

**Figure 1:**
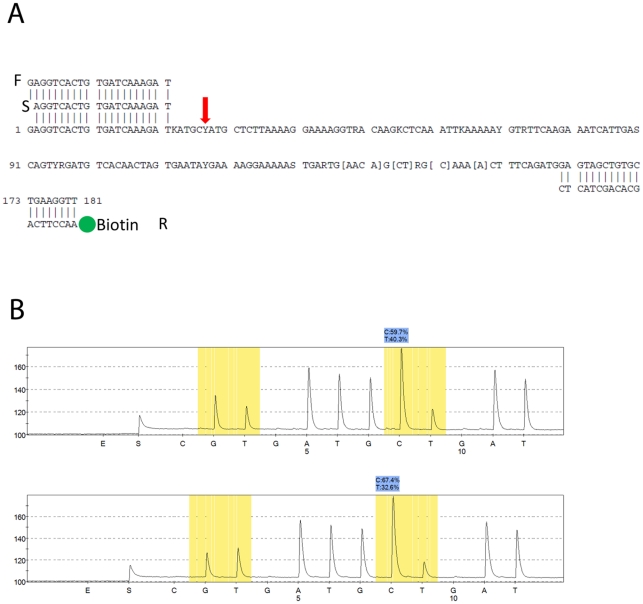
Illustration of the PPRT assay. **A.** Primer design and sequence of region test and reference regions. The sequence shows several differences between chromosome 8 (test) and chromosome 21 (reference). The red arrow show the variable position used to distinguish test from reference amplicon sequences, and quantified by PPRT. F: forward primer, R: reverse primer, and S: sequencing primer. **B.** Pyrograms from PPRT testing of samples with 3 and 4 beta-defensin copies. Two variable sites are highlighted in yellow. The second variable site (corresponding to the site highlighted with the red arrow in Figure 1a), with the percentage of each allele shown, gave reproducible values and used for quantification. For the top pyrogram, the ratio of the C variant (representing the test amplicon) to the T variant (representing the reference amplicon) is 1.5∶1, indicating a diploid copy number of three for the test sequence. For the bottom pyrogram, the ratio is 2∶1, indicating a diploid copy number of four.

PRT was carried out essentially as described previously [50]. The beta-defensin cluster region on chromosome 8 and an identified paralogue gene (*HSPD21* on chromosome 21) 3 kb distal to the *DEFB4* gene with only two copies per genome were PCR amplified. The resulting PCR amplicons differed at 10 positions with an 8 bp (BLAST) difference in length. One of the positions where the amplicons differed was used to quantify the two chromosome regions against each other by pyrosequencing across it. Primers for the pyrosequencing assay were designed using the PSQ assay design software version 1.0.6 (Qiagen, Hilden, Germany). The following sequence was analysed with the position that varied between chromosome 8 and 21 marked with red: KATGC**Y**AT (Figure 1). For the PCR, 20 ng of genomic DNA in a total volume of 50 µl using a forward primer (5′-GAGGTCACTGTGATCAAAGAT-3′) and a reverse primer (5′-Biotin- AACCTTCAGCACAGCTACTC-3′) was used was used together with Q-solution (Qiagen), 10 mM dNTP and Tempase polymerase. PCR was performed on a thermocycler using the following conditions: 15 min at 95°C followed by 35 cycles of 95°C for 30 s, 53°C for 30 s and 72°C for 45 s and one extension step at 72°C for 10 min. The biotin-labeled PCR products were immobilized on streptavidin Sepharose (GE Healthcare, Uppsala, Sweden) by mixing 40 µl of the PCR product with 3 µl streptavidin Sepharose suspension, 40 µl water, and 37 µl 1 x binding buffer (Qiagen). The suspension was shaken at room temperature for 10 minutes. To remove unbiotinylated DNA the samples were sequentially washed in 70% ethanol for 5 seconds, denaturated in 0.5 M NaOH for 5 seconds and additional washing in 1x washing buffer (Qiagen) for 5 seconds. This was done using the PyroMark Vacuum Prep Tool (Qiagen). After the last wash the ssDNA biotinylated DNA was transferred to 39 µl 1 x annealing buffer (Qiagen) and 1 µl sequencing primer (5′-AGGTCACTGTGATCAAAGAT-3′). The suspension was heated to 80°C for 2 minutes and was equilibrated to room temperature for 5 minutes in order to let the sequencing primer anneal. The sequencing reaction was performed using the Pyro Gold Reagent Kit (Qiagen) in the PSQ 96 MA Pyrosequencer (Qiagen) according to the manufacturer's instructions. The relative percentages of the two variants were calculated by the accompanying software and were used for the gene copy number determination. Positive controls with known copy number in each run were used to generate a correction curve by linear regression, and corrected copy number estimates calculated for each sample calculated using this run-specific regression equation. A “no template control” was included in each run.

### Statistical analysis

Statistical analysis was performed using GraphPad InStat 3 (GraphPad Software, La Jolla, USA) and Microsoft Excel.

## Results and Discussion

We have developed a version of the PRT method that uses pyrosequencing and quantification of different sequence variants to distinguish test and reference amplicons (pyrosequencing PRT, PPRT). A pyrosequencing approach to determining beta-defensin copy number has been described previously [42], using different primers and an alternative paralogue on chromosome 5, giving results that differed considerably from MLPA and previously published estimates of the same samples [50]. Nevertheless, given the potential of PPRT to automated copy number typing, and the previously published examples of pyrosequencing to quantify alleles, a reliable PPRT method would be a very useful tool to investigate the role of beta-defensin copy number and disease. We designed an assay that would allow PCR amplification across a “test” region distal to *DEFB4* co-amplifies a “reference” region on chromosome 21. Pyrosequencing of the product allows quantification of a particular sequence variant that reports copy number by distinguishing test from reference amplicons.

**Figure 2:**
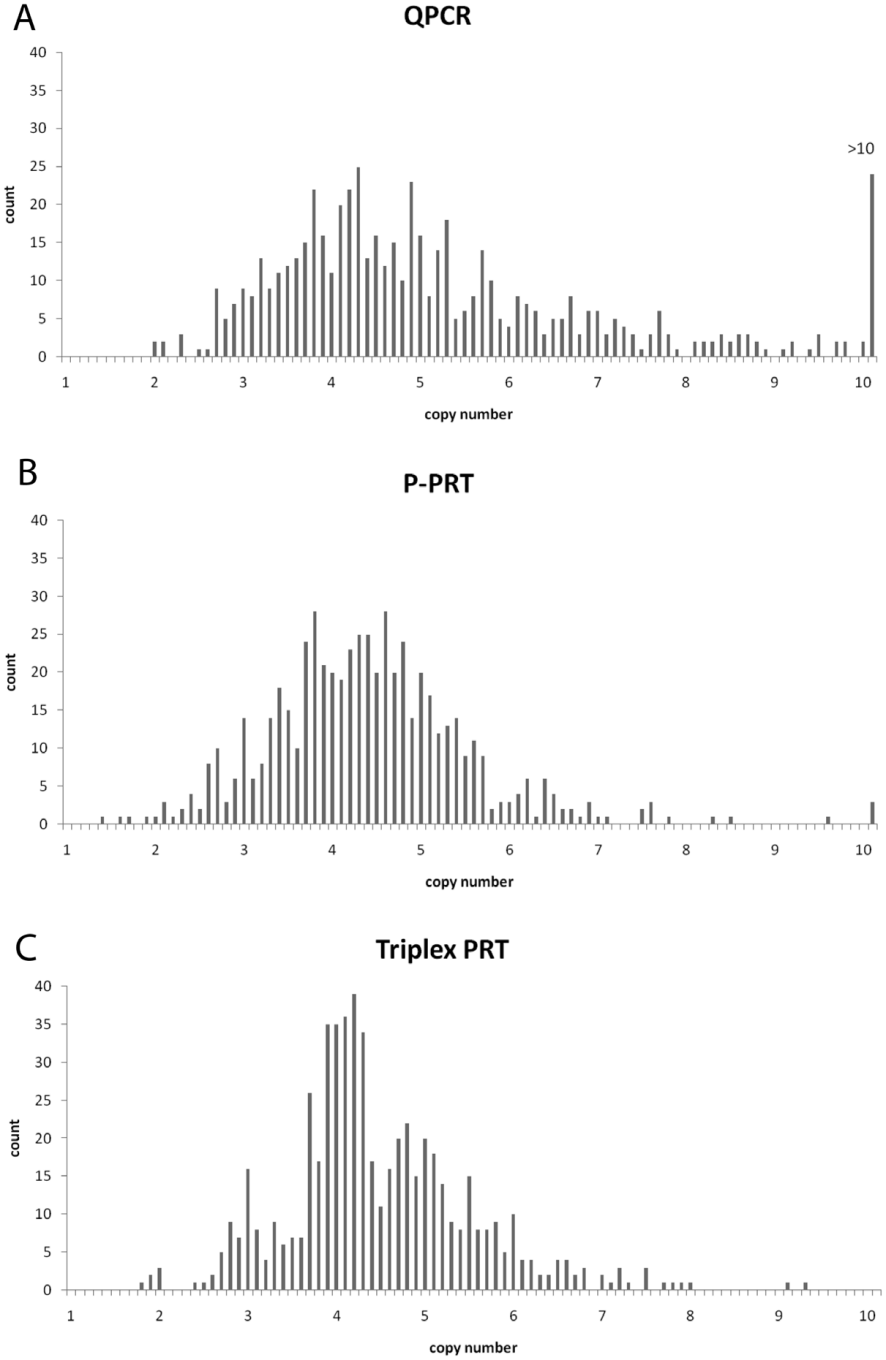
Histograms of raw unrounded copy number estimates. The raw unrounded copy number estimates for all 576 samples analysed. Unrounded copy number estimates are in bins of 0.1, with the count of each bin displayed on the y-axis: a) from QPCR assay, b) from PPRT assay, and c) from triplex PRT assay.

We determined beta-defensin gene copy number using PPRT and two other independent methods on DNA samples from 576 individuals from five populations. Comparison of copy number estimates for 14 samples measured using several different methods shows that triplex PRT and MLPA give exactly the same copy numbers as the previously published PRT method (Table 1). PPRT performs better than a previous pyrosequencing-based assay, but showed three discrepancies with PRT/triplex PRT/MLPA. Extending this analysis, we examined the proportion discordant copy number calls between the three methods (Table 2). There is a very high frequency of discordant calls, although PPRT and triplex PRT are the least discordant (47.3%). Such variation in copy number calling between methods is problematic and is likely to reflect error in the methods used. We decided to investigate the sources of error by examining the raw data in more detail. The raw unrounded data are shown as histograms in Figure 2. If our underlying biological assumption is that copy number varies discretely as complete integers (2, 3, 4 etc) then we would expect our data to reflect that underlying biological reality by clustering of unrounded data about those integer values. Both QPCR and PPRT show no evidence of clustering (Figure 2a, 2b), compared to triplex PRT (Figure 2c), which shows evidence of clustering, revealed by peaks in the histogram corresponding to integer copy numbers. This is due, at least in part, to repeat testing, because each triplex PRT copy number estimate is from duplicate testing of three different assays, compared to triplicate testing of one assay for the QPCR, and one test for the PPRT. Indeed, on repeat testing of a selection of UK samples, the coefficient of variation of PPRT is 0.05 compared to 0.08 for triplex PRT, suggesting that PPRT is as precise as triplex PRT, although PPRT assays a single locus, while the triplex PRT assays three different loci within the beta-defensin CNV.

**Table 1:** Copy number estimates using different methods on a panel of DNA samples.

Sample	PRT (reference 50)	PPRT (this paper)	PPRT (reference 42)	Triplex PRT (this paper)	MLPA (reference 42)	Q-PCR (this paper)
C0088	4	4	3	4	4	4
C0096	5	5	3	5	5	4
C0187	4	4	4	4	4	3
C0195	4	4	4	4	4	4
C0748	4	3	4	4	4	4
C0766	3	2	2	3	3	3
C0863	5	5	4	5	5	5
C0877	3	3	3	3	3	3
C0888	5	5	4	5	5	5
C0909	5	4	4	5	5	6
C0913	3	3	3	3	3	3
C0917	4	4	3	4	4	4
C0937	4	4	4	4	4	4
C0960	3	3	2	3	3	3

This table summarises published data and data from this paper where a panel of DNA samples from the UK population have been typed for beta-defensin copy number by different laboratories using different methods.

**Table 2:** Percentage of discordant results between the three different methods.

	PPRT	Triplex PRT	QPCR
PPRT	-	-	-
Triplex PRT	47.3%	-	-
QPCR	67.7%	60.2%	-

Pairwise discordance rates are shown, for a first-pass test of all 576 samples.

**Figure 3:**
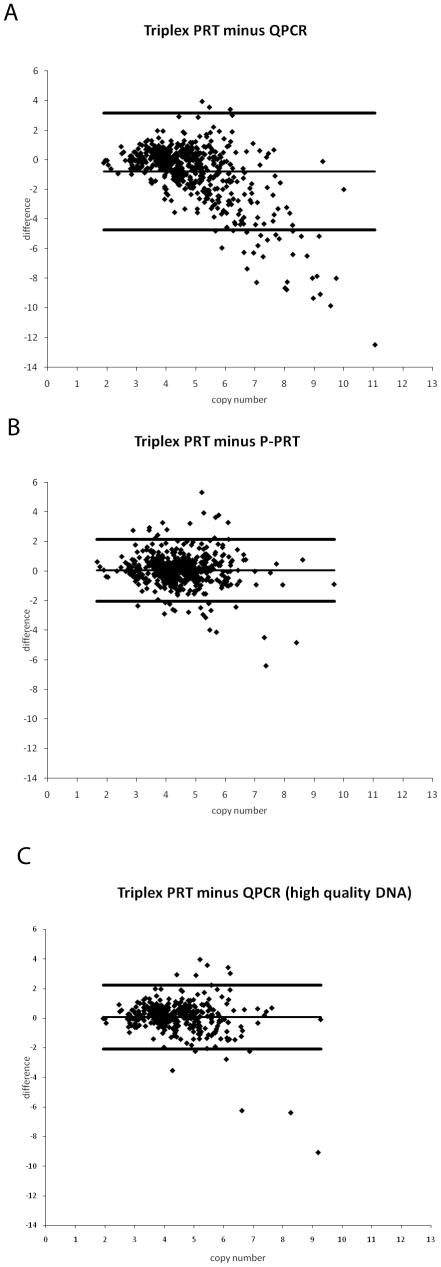
Bland-Altman plots showing differences in copy number estimation between methods. a) Triplex PRT minus QPCR, b) Triplex PRT minus PPRT, c) Triplex PRT minus QPCR, good quality DNA only.

Both PPRT and triplex PRT give a copy number distribution of between 2 and 6 copies, with a few samples showing a higher copy number. However, the QPCR assay gives a considerably broader distribution, with more samples showing apparently higher copy number, and a significant number of samples showing copy number higher than 10. This is reflected in the Bland-Altman plots comparing the raw copy number estimates between the different methods (Figure 3), and the observation that QPCR can overestimate copy number values agrees with other reported studies [55].

**Figure 4:**
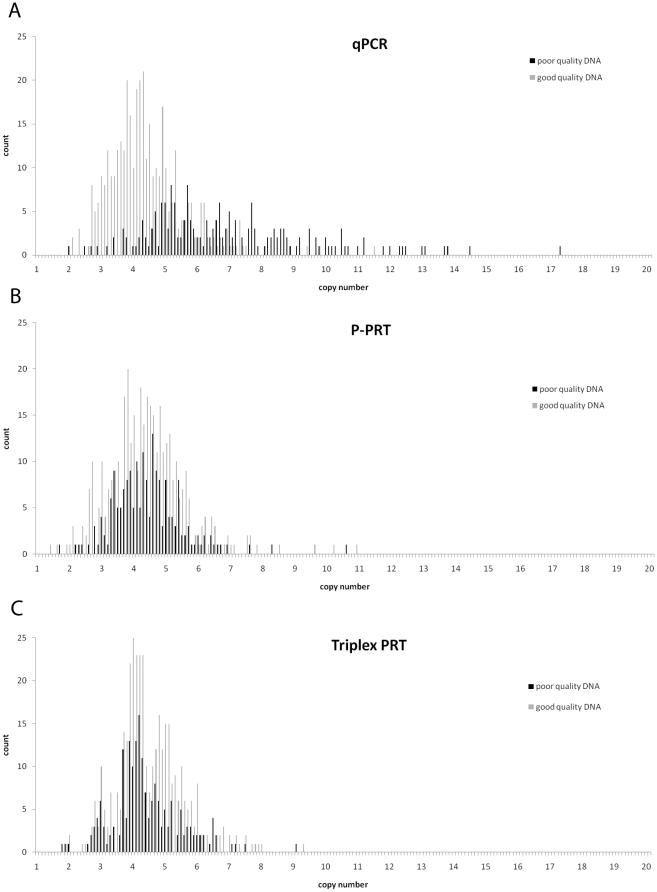
Histograms of raw unrounded copy number estimates comparing results from poor and good quality DNA. The raw unrounded copy number estimates for all 576 samples analysed. Unrounded copy number estimates are in bins of 0.1, with the count of each bin displayed on the y-axis. Results from the good quality DNA cohorts (UK, Danish, Czech) are shown in grey, results from the poor quality DNA cohorts (Portuguese, Ghanaian) are shown in black. a) From QPCR assay, b) from PPRT assay, c) from triplex PRT assay.

**Figure 5:**
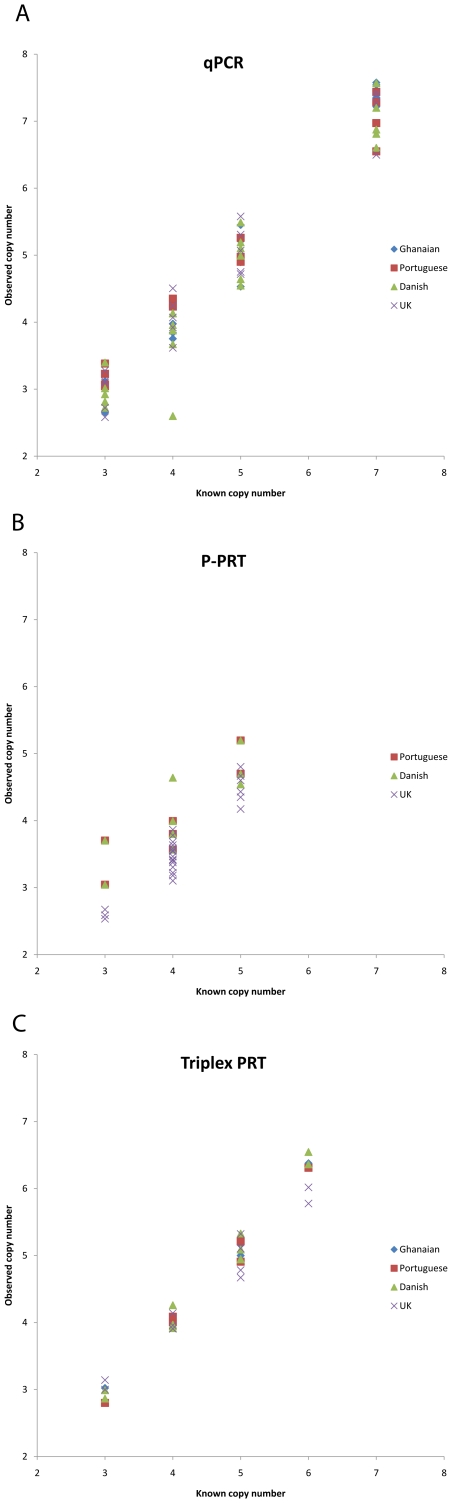
Scatterplots showing reproducibility of positive controls between different experiments. These figures plot known copy number from control samples against actual raw copy number values, for each experiment. Note that all five populations are not represented: several experiments tested samples from more than one population, which was an additional control against systematic bias. The legend on the graphs indicates the population from which the majority of samples in that particular experiment derived. a) QPCR assay, b) PPRT assay, c) Triplex PRT assay.

We took the raw copy number data and divided, according to cohort, by separating the Ghanaian and Portuguese cohorts from the UK, Danish and Czech cohorts. Plotting this raw data as a histogram shows that DNA cohort origin is clearly responsible for systematic copy number calling bias in QPCR between “poor quality” DNA from the Portuguese and Ghanaian cohorts and “good quality” DNA from the UK, Danish and Czech cohorts (Figure 4a) but had no effect on copy number calling for the two PRT-based methods (Figure 4b and 4c) but. For the UK, Danish and Czech cohorts, QPCR gives a copy number distribution in a range comparable with the PRT-based methods, while for the Ghanaian and Portuguese cohorts QPCR systematically overestimates copy number, often by several copies. Because each cohort was analysed as a batch, it is possible that biases in the normalisation to known copy number controls included on every PCR plate (see Methods) could generate this effect. We compared the results from the known copy number controls across each cohort-specific plate for all three methods, and found no evidence of systematic differences (Figure 5).

We therefore reasoned that systematic difference in DNA quality between the DNA sample cohorts may be responsible for this effect. Cukier and colleagues recently showed that degraded DNA could give spurious CNV findings despite the presence of multiplexed internal samples in QPCR assays [45]. Although these five cohorts are from different biological sources, DNA extracted using different methods, and have different histories of transport and storage associated with them, they do not appear to vary with DNA “quality” (Table 3). We investigated whether the systematic bias in QPCR results was due to DNA degradation by analysing selected samples by agarose gel electrophoresis or whether it was due to salt contamination by examining absorbance at 230 nm. Neither measure correlated with the “quality” of the DNA in giving appropriate QPCR results, so we do not yet understand the physical basis for the different DNA qualities of these cohorts.

**Table 3:** Populations analysed and DNA source information.

Population	Number of samples	Biological material	DNA extraction method	DNA quality
**UK**	190	Lymphoblastoid cell line	Magnetic bead affinity	Good
**Denmark**	174	Peripheral blood leukocyte	Qiagen column	Good
**Ghana**	100	Peripheral blood leukocyte	Qiagen column	Poor
**Portugal**	91	Peripheral blood leukocyte	Qiagen column	Poor
**Czech Republic**	21	Peripheral blood leukocyte	“Salting out”	Good

This raises questions about the applicability of QPCR in measuring DNA copy number, particularly as we cannot, as yet, identify the factor that is responsible for this effect, ruling out *a priori* determination of the applicability of each sample for copy number calling by QPCR. Furthermore, it is unclear whether internal controls constructs would remove this effect, given that it is a property of the genomic DNA itself rather than any effect of aberrant normalisation.

Taking the triplex PRT data as representing the correct copy number of the samples typed, we investigated whether there was any significant difference between copy number distributions in the five different populations. There was no significant difference between the means (one-way ANOVA, p>0.05), reflecting essentially no difference between populations within Europe or between European and Ghanaian populations (Table 4). This is consistent with previous studies [39]. This probably reflects the high mutation rate at this locus, causing any population specific signatures to be rapidly erased. This also means that real case-control differences from robust studies are less likely to be confounded by cryptic population stratification than similar SNP studies, although we would recommend that population stratification is controlled for as far as possible.

**Table 4:** Beta-defensin copy number distributions in different populations.

Copy number	UK		Denmark		Ghana		Portugal		Czech Republic	
	count	%	count	%	count	%	count	%	count	%
**2**	2	1	4	2	3	3	2	2	0	0
**3**	26	14	32	19	19	19	11	12	1	5
**4**	88	46	68	40	49	49	41	45	8	38
**5**	53	28	43	25	21	21	28	31	7	33
**6**	16	8	17	10	5	5	7	8	4	19
**7**	3	2	4	2	1	1	1	1	1	5
**8**	1	1	2	1	1	1	1	1	0	0
**9**	1	1	0	0	1	1	0	0	0	0

Beta-defensin copy number, as determined by triplex PRT, are shown for the five populations studied, as counts and as a percentage.

Differences in beta-defensin copy number may have important clinical consequences in the susceptibility to, and progression of, a variety of diseases with an inflammatory or infectious etiology. Accurate and precise methods for measuring copy number are essential when investigating subtle changes in copy number distribution between patients and healthy controls, which may reflect an effect of beta-defensin copy number on susceptibility to the disease under study. In summary, we show that PPRT is a practical high-throughput approach, although we would recommend multiplicate PPRTs per sample are required for sufficient accuracy for case-control analyses, and careful quality-control performed for every experiment. PPRT is cost-effective, with a single test priced around €0.75, compared with around €1.2 for a triplex PRT. We hope that with an increasing focus on reliable methods to type beta-defensin copy number variation we will be in a position to investigate the role of this complex locus in disease.
